# Survival Rate of *Toxoplasma gondii* Tachyzoites in Cow's Milk at Different Temperatures

**DOI:** 10.1002/vms3.70595

**Published:** 2025-08-30

**Authors:** Shadi Khosravi, Nasser Hajipour, Mir‐Hassan Moosavy, Erfan Mosharkesh

**Affiliations:** ^1^ Department of Food Hygiene and Aquatics Faculty of Veterinary Medicine University of Tabriz Tabriz Iran

**Keywords:** cow, East Azerbaijan, heat, milk, *Toxoplasma gondii*

## Abstract

*Toxoplasma gondii* is the causative agent of toxoplasmosis, an obligate intracellular parasite of warm‐blooded animals; the definitive host is cats and felines. Transmission of this parasite in herbivorous intermediate hosts occurs through contaminated water and forage by the oocyst stage of the parasite, and in cats and humans, it occurs through eating contaminated meat and milk by the cystic stage, tachyzoite and oocyst of the parasite. Some people consume the milk of various animals, including cows, sheep, goats, camels and donkeys. Such kinds of milk are supposed to be a good source of protein and vital minerals. Nonetheless, they are frequently ingested uncooked or not heated with high temperatures to destroy dangerous microbes. Hence, the potential viability of *T. gondii* tachyzoites in the milk of these animals under various temperatures needs to be investigated, as this could be a significant risk indicator of human infections. For this purpose, purchase 1000.0 mL of cow's milk from milk supply centres and then divide it into different parts, and after adding *T. gondii* tachyzoites of the RH strain (10^7^ × 5) to each milk sample, they are subjected to rapid pasteurization temperatures (75°C for 15 s), slow pasteurizations (60°C for 15 min), temperatures of 25°C and 35°C for 3, 6 and 12 h and refrigerator temperatures (4°C for 6, 12 and 24 h), and after centrifugation, the resulting precipitate was injected three times into three mice. One mouse without parasite tachyzoite injection was considered a negative control, and three mice injected with parasite tachyzoites were considered as positive controls. Our results showed that in the first trial, *T. gondii* tachyzoites treated at 75°C, 60°C, 35°C, 25°C and 4°C survived after 15 s and caused the death of mice. However, in the second trial, at 75°C, the parasite tachyzoites were completely eliminated after both 15 min and 3 h, and all treated mice survived. In contrast, in the third trial, at 4°C, 25°C and 35°C, after 15 min, 3, 6, 12 and 24 h, the parasite survived and caused the death of mice. Statistical analysis showed that the mortality of mice treated at 75°C and 60°C during the treatment periods was significant. This study showed that milk possibly contaminated with parasitic tachyzoites can survive in rapid and slow pasteurization and refrigerator temperatures, suggesting heating milk at 60°C for more than 15 min is recommended.

## Graphical abstract

The research paper identifies the thermal resistance of *Toxoplasma gondii* tachyzoites in cow's milk at different temperatures. The results indicate the possible danger of being infected by the raw or poorly heated milk, which suggests a great emphasis on the importance of thermal treatment of food to guarantee its safety.

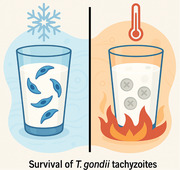

## 1 Introduction

*Toxoplasma gondii* is a globally distributed obligate intracellular protozoan parasite capable of infecting all warm‐blooded animals including humans. The primary host for sexual reproduction of this parasite is the domestic cat and other felids, which excrete environmentally resistant oocysts in their faeces. The infection is acquired by an intermediate host, such as humans, through ingestion of sporulated oocysts contained in contaminated food, water or soil, or from the consumption of meat or dairy products containing viable tissue cysts or tachyzoites. Immunocompetent individuals usually exhibit an asymptomatic form of toxoplasmosis, whereas immunocompromised patients and foetuses are at risk for serious complications of toxoplasmosis, including encephalitis, retinochoroiditis and congenital malformations. Therefore, *T. gondii* remains a significant public health concern and an important foodborne zoonotic disease (Torgerson and Mastroiacovo 2013; Vilela and Feitosa 2024; Galván‐Ramírez et al. 2024).

Historically, the transmission of *T. gondii* has been considered to occur via ingestion of undercooked meat, most typically from infected sheep or pigs. But recent work establishes that unpasteurized milk is an underappreciated but potent route of infection. Milk and dairy products are consumed globally and simply include milk, cheese, butter, yogurt and others that can be helpful to the welfare in terms of protein, calcium and some other nutrients. In many cases, milk from cows, sheep, goats and camels, as well as donkey's milk, is consumed raw or heated slightly due to cultural preference or absence of appropriate pasteurization infrastructure, particularly in rural and lower‐income areas. It is possible exposure to viable *T. gondii* tachyzoites in dairy milk during the storage and illumination of the milk containers with ultraviolet light when these conditions are met (Atif et al. 2024; Yücesan 2024; Ait Issad et al. 2020).

The presence of *T. gondii* DNA in raw milk samples from different animal species has been confirmed by recent molecular and experimental studies. In a study performed by Alipour Amroabadi et al. (2020), it was found that the B1 gene of *T. gondii* was detected in 6.1% of raw cow milk samples and 10.0% of sheep milk samples collected from local markets and traditional dairies in Iran, suggesting a high risk of exposure. Other similar studies have also reported the presence of viable tachyzoites in goat and camel milk, pointing to the possibility of a zoonosis from unprocessed milk. Viable tachyzoites isolated from milk were shown to be capable of causing fatal toxoplasmosis in experimental models including Bagg Albino and severely combined immunodeficient mice in immunological and animal infectivity assays, as evidence of infectivity and resistance to mild environmental stressors (Alipour Amroabadi et al. 2020).

The survival and ability to infect of *T. gondii* tachyzoites depend on temperature conditions while also being affected by the duration of suspension and the chemical components in their environment. Nguyen et al. (2022) researched tachyzoite viability using different nutrient solutions exposed to 3°C–9°C refrigeration and 25°C room temperature environments, which confirmed that tachyzoites remained alive for up to 6 h. Survival prospects of *T. gondii* in milk kept under home refrigerator temperatures and open‐air market conditions in residential settings remain a cause for safety concern (Nguyen et al. 2022). Procedures such as standard milk pasteurization at 72°C for 15 s proved inadequate to eliminate *Encephalitozoon cuniculi* protozoan parasites, according to Kváč et al. (2016) while showing that thermal resistance spans across different protozoan species and necessitates further assessment of pasteurization standards for milk protozoa contamination (Kváč et al. 2016).

The global community adopts pasteurization as the superior treatment method for microbial pathogen elimination in milk through the use of either HTST (72°C–75°C for 15 s) or LTLT (60°C–65°C for 15–30 min) heat processes. Research shows that protozoan parasite survival happens during suboptimal pasteurization procedures or heating variations, mostly in milk processing that involves large amounts of milk and unsophisticated domestic heating equipment. Rani and Pradhan (2021) estimated *T. gondii* safety parameters in cooked meat by using Monte Carlo modelling, which showed that 62.8°C temperatures allowed only minimal cyst survival, which increased researchers' concerns about milk matrix thermal resistance (Rani and Pradhan 2021).

The survival patterns of *T. gondii* tachyzoites in cow's milk under various temperature–time conditions remain poorly documented despite growing scientific evidence. The scarcity of data about tachyzoites' survival in cow's milk poses a significant concern because milk remains widely used in industrial and traditional dairy industries. Previous research primarily investigated *T. gondii* survival in meat products, as well as tissue cysts and animal host serological detection. A limited number of scientists have researched tachyzoite survival patterns in milk, as they multiply rapidly and transmit the infection efficiently under typical consumer milk storage and handling environments (Nguyen et al. 2022; Holec‐Gąsior and Sołowińska 2023).

This research explores the survival patterns of *T. gondii* tachyzoites in cow's milk exposed to different heat treatments with rapid pasteurization (75°C for 15 s) and slow pasteurization (60°C for 15 min) and storage at refrigeration (4°C), ambient (25°C) and elevated room temperatures (35°C) across 15‐s to 24‐h time intervals. A murine bioassay serves as our model to evaluate the vitality of tachyzoites through exposure scenarios that we measure against infection potential. This research investigation is set to generate findings about *T. gondii* tachyzoite thermal resistance in milk which will support public health guidelines and food safety procedures, particularly in areas where people continue to consume unpasteurized milk.

## 2 Methods and Materials

### 2.1 Study Design

In this research, to obtain cow's milk samples, collection centres in Tabriz County, East Azerbaijan Province, were visited, and 1000.0 mL of milk was purchased. The milk was immediately transferred to the Food Hygiene Laboratory of the Faculty of Veterinary Medicine at the University of Tabriz and stored in a refrigerator until the time of analysis. Additionally, 40 Swiss mice weighing 30 ± 20 g were purchased from the Faculty of Pharmacy at Urmia University of Medical Sciences and transferred to the animal house of the University of Tabriz in cages. All experimental procedures involving animals were approved by the Biomedical Ethics Committee of the University of Tabriz, under the approval letter dated 2 July 2025. The study was conducted in full compliance with the institutional guidelines for the care and use of laboratory animals.

### 2.2 Preparation of *T. gondii* Tachyzoites

Tachyzoites of the *T. gondii* RH strain were obtained from the Parasitology Department of the Faculty of Medicine at the University of Tabriz and intraperitoneally injected into a number of Swiss mice. After 3 days, when the parasite reached its maximum replication, aspiration of the parasites from the peritoneal cavity of the mice was initiated. For this purpose, the Swiss mice were individually anaesthetized with ether, and after intraperitoneal injection of 1.0–2.0 mL of physiological saline and massaging each mouse, the parasitic tachyzoites were aspirated using an insulin syringe. The aspirated tachyzoites were collected in a sterile Falcon tube and stored in a refrigerator until the experiment was conducted (Dahmane et al. 2025; Arruda et al. 2024; Nguyen Thi et al. 2022).

### 2.3 Main Process

To investigate the effect of flash pasteurization temperature (75°C) on the viability of *T. gondii* tachyzoites in milk (Nguyen Thi et al. 2022), 10.0 mL of milk was poured into a test tube, and 5 × 10^5^ *T. gondii* RH strain tachyzoites were injected into the milk‐containing test tube using a syringe. Prior to this, the temperature of a water bath was set at 75°C. The test tube containing the milk treated with the parasite was placed in the water bath, and the temperature inside the test tube was monitored with a thermometer. As soon as the temperature reached 75°C, the water bath was covered, and the sample was held at this temperature for 15 s. After this time, the test tubes were removed and centrifuged at 2000.0 rpm for 5 min. Finally, the resulting pellet was resuspended and intraperitoneally injected into three mice with three repetitions. These mice were kept in cages for 7 days, and the injection and death dates of the mice were recorded. Three mice that were intraperitoneally injected with *Toxoplasma* tachyzoites served as a positive control, and one mouse without injection was considered a negative control.

To investigate the effect of slow pasteurization temperature (60°C) on the viability of *T. gondii* tachyzoites (Nguyen Thi et al. 2022), 10.0 mL of milk was pipetted into a test tube, and 5 × 10^5^ *T. gondii* RH strain tachyzoites were injected into the milk‐containing test tube using a syringe. Prior to this, the temperature of a water bath was set at 60°C. The test tube containing the milk treated with the parasite was placed in the water bath, and the temperature inside the test tube was monitored with a thermometer. As soon as the temperature reached 60°C, the water bath was covered, and the sample was held at this temperature for 15 min. After this time, the test tubes were removed and centrifuged at 2000.0 rpm for 5 min. Finally, the resulting pellet was resuspended and intraperitoneally injected into three mice with three repetitions. These mice were kept in cages for 7 days, and the injection and death dates of the mice were recorded. Three mice that were intraperitoneally injected with *Toxoplasma* tachyzoites served as a positive control, and one mouse without injection was considered a negative control.

To investigate the effect of 25°C and 35°C temperatures over durations of 3, 6 and 12 h on the viability of *T. gondii* tachyzoites, 10.0 mL of milk was pipetted into test tubes, and 5 × 10^5^ *T. gondii* RH strain tachyzoites were injected into the milk‐containing test tubes using a syringe (Nguyen Thi et al. 2022).

Prior to this, the temperatures of two separate water baths were set at 25°C and 35°C. The test tubes containing the milk treated with the parasite were placed in the respective water baths, and the temperature inside each test tube was monitored with a thermometer. Upon reaching the target temperature of 25°C (for one set of tubes) and 35°C (for the other set), the water baths were covered, and the samples were incubated for durations of 3, 6 and 12 h.

After these incubation periods, the test tubes were removed and centrifuged at 2000.0 rpm for 5 min. Finally, the resulting pellet was resuspended and intraperitoneally injected into three mice with three repetitions. These mice were kept in cages for 7 days, and the injection and death dates of the mice were recorded. Three mice that were intraperitoneally injected with *Toxoplasma* tachyzoites served as a positive control, and one mouse without injection was considered a negative control. The procedure for investigating the effect of 35°C on the viability of *T. gondii* tachyzoites in milk over durations of 3, 6 and 12 h was identical to the method described above (Nguyen Thi et al. 2022).

### 2.4 Analysis of Results

The obtained data were analysed using SPSS statistical software (version 21) with a one‐way ANOVA test at a 95.0% confidence level. In cases where the difference between means was significant, Duncan's post hoc test was used to further investigate the differences between the groups. A *p*‐value of less than 0.05 (*p *< 0.05) was considered statistically significant.

## 3 Results

The results of this study indicated that when *T. gondii* tachyzoites treated at 75°C for 15 s were injected into three mice, all three died. This suggests that the parasite tachyzoites added to the milk and treated at 75°C for 15 s remained viable and caused mortality in the Swiss mice following intraperitoneal injection. However, when the pellet obtained from centrifuging the milk contaminated with tachyzoites and treated at the same temperature for 15 min was injected into three mice, only one mouse died after 7 days. Furthermore, all three mice survived when injected with the pellet from milk treated at 75°C for 3 h. In other words, the parasite tachyzoites exposed to 75°C in milk for 3 h were no longer viable after centrifugation, and therefore, the mice survived. Since the parasite tachyzoites were non‐viable at 75°C after 3 h, resulting in the survival of all three mice, further experiments at 12 and 24 h were not conducted.

The results showed that when the pellet obtained from centrifuging milk contaminated with *T. gondii* tachyzoites and treated at 4°C for 15 s, 15 min, 3, 6, 12 and 24 h was injected into three mice, it caused their death within 2–5 days. These findings indicate that the parasite remained viable at the specified temperature and durations, leading to the death of the Swiss mice. Similarly, at 25°C, *T. gondii* treated for 15 s, 15 min, 3, 6, 12 and 24 h caused mortality in the injected mice within 3–7 days. Furthermore, *T. gondii* treated at 35°C for the same durations (15 s, 15 min, 3, 6, 12 and 24 h) resulted in the death of the injected mice within 3–8 days. At 60°C, *T. gondii* treated for 15 s and 15 min led to the death of the mice, but the parasite did not survive treatments of 3, 6, 12 and 24 h, and all mice in these groups survived (Table 1).

**TABLE 1:** Effect of different temperatures and exposure times on the mortality of mice injected with *Toxoplasma gondii*‐contaminated milk.

Temperature (°C)	Exposure time	Number of mice	Number of deaths	Mortality rate (%)	Mean time to death (days ± SD)
4	15 s	3	3	100	2.0 ± 0.0
4	15 min	3	3	100	2.0 ± 0.0
4	3 h	3	3	100	3.0 ± 0.0
4	6 h	3	3	100	3.0 ± 0.0
4	12 h	3	3	100	4.0 ± 0.6
4	24 h	3	3	100	5.0 ± 0.0
25	15 s	3	3	100	4.0 ± 0.0
25	15 min	3	3	100	3.0 ± 0.0
25	3 h	3	3	100	13.0 ± 5.3
25	6 h	3	3	100	6.0 ± 1.0
25	12 h	3	3	100	3.0 ± 0.0
25	24 h	3	3	100	6.0 ± 4.4
35	15 s	3	3	100	3.0 ± 0.0
35	15 min	3	3	100	3.3 ± 0.6
35	3 h	3	3	100	6.0 ± 3.5
35	6 h	3	3	100	4.7 ± 1.2
35	12 h	3	3	100	4.7 ± 2.1
35	24 h	3	3	100	4.7 ± 1.2
60	15 s	3	3	100	3.0 ± 0.0
60	15 min	3	1	33.3	7.0 ± N/A
60	3 h	3	0	0	N/A
60	6 h	3	0	0	N/A
60	12 h	3	0	0	N/A
60	24 h	3	0	0	N/A
75	15 s	3	3	100	3.0 ± 0.0
75	≥ 15 min	—	—	—	—

*Note*: Data for the 75°C treatment group at exposure times of 15 min, 3, 6, 12 and 24 h were not available for this analysis.

The results of the statistical analysis, as indicated by the survival or death of the Swiss mice following injection with *T. gondii* tachyzoites treated at different temperatures in cow's milk, revealed a significant correlation between the mortality rate of mice and the different time durations at 75°C and 60°C, while the correlation was non‐significant at 4°C, 25°C and 35°C across the tested time points (Table 2).

**TABLE 2:** Statistical analysis of the association between mortality in injected mice and sediment from centrifuged milk contaminated with *Toxoplasma gondii* tachyzoites treated at different temperatures.

Time/temperature (°C)	4°C	25°C	35°C	60°C	75°C
15 s	*p* > 0.05	*p* > 0.05	*p* > 0.05	*p* < 0.05	*p* < 0.05
15 min	*p* > 0.05	*p* > 0.05	*p* > 0.05	*p* < 0.05	*p* < 0.05
3 h	*p* > 0.05	*p* > 0.05	*p* > 0.05	*p* < 0.05	*p* < 0.05
6 h	*p* > 0.05	*p* > 0.05	*p* > 0.05	*p* < 0.05	*p* < 0.05
12 h	*p* > 0.05	*p* > 0.05	*p* > 0.05	*p* < 0.05	*p* < 0.05
24 h	*p* > 0.05	*p* > 0.05	*p* > 0.05	*p* < 0.05	*p* < 0.05

## 4 Discussion

As previously mentioned, zoonotic diseases shared between humans and animals represent a significant public health concern globally, particularly in developing countries. These diseases are important in terms of human health, animal health, economics and disease control efforts. *T. gondii* is one such foodborne zoonotic parasite, including transmission through milk, and has been reported in various herbivorous animals. Humans can become infected by consuming milk and meat contaminated with the tachyzoite form of the parasite or by ingesting food contaminated with the oocyst form.

The results of our research demonstrated that *T. gondii* tachyzoites treated at 75°C for 15 s and at 4°C, 25°C and 35°C for durations of 15 s, 15 min, 3, 6, 12 and 24 h remained viable and caused mortality in mice. Furthermore, treatment at 60°C for 15 s and 15 min also resulted in the death of mice. According to the present study, at temperatures of 75°C and 60°C, since the *p*‐value is < 0.05, a significant relationship exists between temperature and time. However, at temperatures of 4°C, 25°C and 35°C, given that the *p*‐value is > 0.05, there is no significant relationship between temperature and time.

A study done recently in Brazil found *T. gondii* DNA in 2.8% of the raw milk samples taken from actual lactating cows. Curiously, the serum of the cows whose milk tested positive for *T. gondii* DNA was seronegative for anti‐*T. gondii* IgG antibodies and indicated the possibility of shedding of parasites during the acute phase of infection. The presence of parasite DNA in milk does not conclusively prove the existence of viable and infectious tachyzoites in the milk, but is of such great public health concern that the consumption of unpasteurized milk is a major concern (da Fonseca et al. 2023). The implication of these findings is to call for additional research into the infectivity of *T. gondii* in raw milk and, particularly, of the risk related to transmission by milk in endemic areas.

In a study by Nguyen et al. (2022), they investigated whether *T. gondii* tachyzoites are viable in temperatures and nutrient media. However, their findings showed that tachyzoites retain viability up to 6 h of refrigeration (3°C–9°C) and room temperature (25°C), especially when in nutrient‐rich environments like foetal bovine serum. The study also showed a progressive decline in tachyzoite viability in response to longer exposure time and temperature, as well as the possibility for survival for a few hours if there is no storage for raw milk conditions. The results of our study and these observations support the conclusion that milk *T. gondii* tachyzoites remain infectious enough to pose a public health risk for consumers of milk under nonthermal storage conditions such as refrigeration and ambient temperature (Nguyen et al. 2022). As a result, heat treatment of milk to appropriate levels before use becomes even more important, particularly where raw milk is routinely consumed.

Rani and Pradhan (2021) investigated the relationship between time and temperature on the survival rate of *T. gondii* during the cooking and low‐temperature storage of fresh‐cut meats. They employed various statistical sampling techniques, such as bootstrap resampling and Gibbs sampling, to establish this relationship and utilized Monte Carlo simulation to estimate safe temperatures for cooking and storing meat. The results indicated that *T. gondii* was not detected in fresh meats when the internal temperature reached above 4°C and below 18°C. Tissue cysts could survive for at least 30 days at 4°C, and approximately 3.3% of the cysts survived at 62.8°C (Rani and Pradhan 2021).

Research conducted by Franssen et al. (2019) on the effectiveness of treatments on food of animal origin demonstrated that cooking at a core temperature of 60°C–70°C for 15–30 min inactivates parasites in most matrices. Freezing at −21°C for 1–7 days generally inactivates parasites in food of animal origin; however, this cannot be reliably depended upon in domestic settings. Parasitic stages are sensitive to 2.0%–5.0% NaCl, with sensitivity often increased by a reduction in pH. Gamma irradiation at doses greater than 0.5–1.0 kGy is effective against fish parasites (with the exception of Anisakis, which requires 10.0 kGy) (Franssen et al. 2019).

Kváč et al. (2016) demonstrated that *E. cuniculi* in raw cow's milk remains infectious to severely combined immunodeficient mice after pasteurization. Using nested polymerase chain reaction (PCR), 1 out of 50 lactating cows persistently shed *E. cuniculi* in their faeces and milk. Under experimental conditions, *E. cuniculi* spores in milk remained infectious to SCID mice following pasteurization treatments at 72°C for 15 s or 85°C for 15 s (Kváč et al. 2016).

Alipour Amroabadi et al.'s (2020) study on the prevalence of *T. gondii* in 370 samples of raw milk and traditional dairy products collected showed that the specific B1 gene of *T. gondii* was positive in 18 samples out of 370 samples (4.9%). The molecular incidence of *T. gondii* in this study regarding raw cow's milk was 6.1%. *T. gondii* was also observed in 2.9% of traditional dairy samples studied. Sheep's milk (10.0%) and cheese (6.6%) had the highest incidence of parasites. The molecular incidence of *T. gondii* in raw camel milk and cream and butter samples was 5.0%, 5.0% and 3.3%, respectively. The samples collected in autumn (15.6%) and summer (9.4%) had the highest molecular incidence of *T. gondii* (Alipour Amroabadi et al. 2020).

## 5 Conclusion

Our research demonstrates that tachyzoites maintain survival abilities across temperatures from refrigeration levels at 4°C to ambient temperatures at 25°C and room conditions at 35°C during a 24‐h period. The 75°C heat treatment for 15 min successfully reduced parasite viability, but further reduction in heat duration at 75°C or heated retention at 60°C for 15 min failed to achieve complete inactivation, as confirmed through mouse model mortality.

Statistics show that higher temperature levels (60°C and 75°C) demonstrate increased associations between parasite viability and heat exposure, while lower temperatures fail to show such associations. Domestic milk‐handling procedures fail to sufficiently remove protozoan pathogens, as seen by the study data from milk‐selling areas that consume raw milk products. Thus, these data reject the belief that brief milk heating can guarantee pathogen elimination.

The proper adherence to heat processing techniques remains essential for protecting consumers against global toxoplasmosis threats found in domestic and artisanal dairy production facilities. In order to confirm the complete destruction of *T. gondii* tachyzoites, milk needs to undergo 60°C heat treatment exceeding 15 min, while exposure to 75°C heat for at least 3 min also confirms clear zygote annihilation. The research reveals that public health education and strict food safety practices must receive increased attention within developing world locations because these areas lack industrial pasteurization capabilities.

Further studies also need to address other environmental factors, such as pH, salt concentration and fat content, that affect tachyzoites' survival in milk. In addition, survival studies of oocysts and bradyzoites will be helpful in understanding various types of dairy products. This will assist in fine‐tuning the control measures and further alleviating the burden due to milk‐borne toxin transmission.

## Author Contributions

S.K. contributed in performing study, sampling, laboratory metabolites analysis, data collection and analysis, writing – review and editing; M.‐H.M. contributed in conceptualization, supervision for the research activities, funding resources, verification of data, data collection and analysis, design of methodology; N.H. contributed in data curation, design of methodology, validation, Investigation; and E.M. contributed in investigation, writing of the original draft and writing and editing of the manuscript. All authors reviewed the manuscript. The authors read and approved the final manuscript being submitted.

## Ethics Statement

The legal requirements or guidelines in our country and province for the care and use of animals have been followed.

## Consent

The authors have nothing to report.

## Conflicts of Interest

The authors declare no conflicts of interest.

## Peer Review

The peer review history for this article is available at https://www.webofscience.com/api/gateway/wos/peer‐review/10.1002/vms3.70595.

## Data Availability

The authors have nothing to report.
